# Platelet‐rich fibrin suppresses in vitro osteoclastogenesis

**DOI:** 10.1002/JPER.19-0109

**Published:** 2019-09-17

**Authors:** Zahra Kargarpour, Jila Nasirzade, Franz Josef Strauss, Francesca Di Summa, Sadegh Hasannia, Heinz‐Dieter Müller, Reinhard Gruber

**Affiliations:** ^1^ Department of Oral Biology Medical University of Vienna Vienna Austria; ^2^ Department of Biochemistry Faculty of Biological Sciences Tarbiat Modares University Tehran Iran; ^3^ Department of Conservative Dentistry School of Dentistry University of Chile Santiago Chile; ^4^ Department of Periodontology School of Dental Medicine University of Bern Bern Switzerland

**Keywords:** alveolar ridge augmentation, bone resorption, osteoclasts, platelet‐rich fibrin

## Abstract

**Background:**

Platelet‐rich fibrin (PRF) membranes can preserve alveolar ridge dimension after tooth extraction. Thus, it can be presumed that PRF suppresses the catabolic events that are caused by osteoclastic bone resorption.

**Methods:**

To address this possibility, we investigated the impact of soluble extracts of PRF membranes on in vitro osteoclastogenesis in murine bone marrow cultures. Osteoclastogenesis was induced by exposing murine bone marrow cultures to receptor activator of nuclear factor kappa B ligand (RANKL), macrophage colony‐stimulating factor (M‐CSF) and transforming growth factor‐beta 1 (TGF‐β1) in the presence or absence of PRF. Osteoclastogenesis was evaluated based on histochemical, gene expression, and resorption analysis. Viability was confirmed by formation of formazan crystals, live‐dead staining and caspase‐3 activity assay.

**Results:**

We report here that in vitro osteoclastogenesis is greatly suppressed by soluble extracts of PRF membranes as indicated by tartrate‐resistant acid phosphatase (TRAP) staining and pit formation. In support of the histochemical observations, soluble extracts of PRF membranes decreased expression levels of the osteoclast marker genes TRAP, Cathepsin K, dendritic cell‐specific transmembrane protein (DCSTAMP), nuclear factor of activated T‐cells (NFATc1), and osteoclast‐associated receptor (OSCAR). PRF membranes, however, cannot reverse the process once osteoclastogenesis has evolved.

**Conclusion:**

These in vitro findings indicate that PRF membranes can inhibit the formation of osteoclasts from hematopoietic progenitors in bone marrow cultures. Overall, our results imply that the favorable effects of PRF membranes in alveolar ridge preservation may be attributed, at least in part, by the inhibition of osteoclastogenesis.

## 1 INTRODUCTION

Platelet‐rich fibrin (PRF), an autologous preparation of coagulated plasma, was originally introduced to improve wound healing.^1^ Today, however, emerging evidence from randomized controlled trials suggests that PRF can preserve horizontal and vertical ridge dimension after tooth extraction.^2^, ^3^, ^4^ Further, preclinical studies support the use of PRF in bone regeneration. For example, in rat craniofacial defects PRF considerably increased bony coverage compared to empty defects.^5^, ^6^ Although there is conflicting evidence for the effect of PRF in terms of bone regeneration,^4^, ^7^ the aforementioned findings provide a scientific basis to raise the hypothesis that PRF helps to maintain the dimension of the alveolar bone by reducing the formation of the bone‐resorbing osteoclasts. It therefore becomes an open question whether PRF affects the process of osteoclastogenesis.

Osteoclasts originate from hematopoietic progenitors. In the presence of survival factor macrophage colony‐stimulating factor (M‐CSF) and the differentiation factor receptor activator of nuclear factor kappa B ligand (RANKL) these progenitors become multinucleated cells termed osteoclasts, staining positive for tartrate‐resistant acid phosphatase (TRAP).^8^ Osteoclasts are further characterized by the expression of genes that are up‐regulated during their differentiation. The genes critical for osteoclastogenesis include Cathepsin K and TRAP, along with the transcription factors nuclear factor of activated T‐cells (NFATc1), regulating costimulatory molecules such as osteoclast‐associated receptor (OSCAR),^9^ and the cell fusion gene dendritic cell‐specific transmembrane protein (DCSTAMP).^10^

Supernatants from activated purified platelets support osteoclastogenesis^11^ whereas preparations containing plasma components decrease osteoclastogenesis in bone marrow cultures rich in hematopoietic progenitors.^12^ Recent evidence suggests that PRF and calcium phosphate decrease the formation of TRAP‐positive cells originating from human peripheral blood mononuclear cells.^13^ This decrease was explained by PRF‐induced cell apoptosis.^13^ Peripheral blood mononuclear cells, however, are not hematopoietic progenitors and genes representing osteoclast function and differentiation remain to be determined. Thus, it is unclear whether PRF reduces osteoclastogenesis in traditional murine bone marrow cultures, rich in hematopoietic progenitor cells.^14^ Consequently, there is a clear demand to refine current knowledge of the impact of PRF on osteoclastogenesis.

In an effort to elucidate the cellular and molecular mechanism by which PRF supports ridge preservation we tested the hypothesis that PRF reduces the formation of osteoclasts in murine bone marrow cultures.

## 2 MATERIALS AND METHODS

### 2.1 Preparation of PRF membranes

PRF membranes were prepared after the approval by the ethics committee of the Medical University of Vienna (1644/2018), and volunteers signed informed consent. All experiments were performed in accordance with relevant guidelines and regulations and were conducted in accordance with the Helsinki Declaration of 1975, as revised in 2013. Venous blood was collected at the University Clinic of Dentistry from six healthy volunteers, each donating six 10 mL plastic glass‐coated tubes^1^ allowing spontaneous blood coagulation. PRF membranes were produced using a protocol of 1570 RPM for 12 minutes (RCF‐max = 400 g). PRF membranes were produced using a centrifuge device^2^ with universal swing‐out rotors (146 mm at the max). The PRF clot was separated from the remaining red thrombus and compressed between two layers of dry gauze. Each PRF membrane was transferred into serum‐free medium (1 cm PRF/mL) and exposed to two cycles of freeze‐thawing and sonication^3^ as reported for human platelet lysate.^15^, ^16^, ^17^ After centrifugation at 15,000 × *g* for 10 minutes, the supernatants of the PRF membranes were harvested and stored at −20°C before the in vitro analysis. In indicated experiments, PRF membranes were transferred into serum‐free medium (1 cm PRF/mL) and placed into an incubator at 37°C to allow a natural release of growth factors into the culture media, similarly as previously described.^18^ At 24 and 72 hours the conditioned medium was collected.

### 2.2 In vitro osteoclastogenesis in bone marrow cultures

BALB/c mice, 6 to 8 weeks old, were purchased from Animal Research Laboratories, Himberg, Austria. Bone marrow cells were collected from the femora and tibiae of the mice as previously described.^19^ Bone marrow cells were seeded at 4 × 10^6^ cells/cm^2^ into 24‐well plates and grown for 5 days in Minimum Essential Medium Eagle‐Alpha Modification^4^ (αMEM) supplemented with 10% fetal calf serum (FCS)^5^ and 1% antibiotics.** Receptor activator of nuclear factor kappa‐B ligand (RANKL, 30 ng/mL),^6^ macrophage colony‐stimulating factor (M‐CSF, 20 ng/mL),^‡‡^ and human transforming growth factor beta 1 (TGF‐β1, 10 ng/mL)^‡‡^ were used to induce osteoclastogenesis. If not otherwise indicated, 50% PRF was included in the culture medium. After 5 days, histochemical staining for tartrate‐resistant acid phosphatase (TRAP)^7^ was performed following the instructions of the manufacturer. Cells with three or more nuclei were counted positive for osteoclasts. For pit formation, osteoclastogenesis was performed on the surface of bovine dentine slices. After 7 days cells were removed and the dentine slices were stained with 1% toluidine blue.

### 2.3 RT‐PCR

At day 5 of bone marrow culture, total RNA was isolated^8^ and reverse transcription (RT) was performed.^9^ RT‐PCR was done using the manufacturer's instructions.^##,^ 10 Primer sequences are given in Supporting Information Table 1 in online *Journal of Periodontology*. Relative gene expression was calculated with the delta delta CT method using a software.^11^ Reactions were run in duplicates.^12^

### 2.4 Cell viability, proliferation, and caspase‐3 activity assay

Bone marrow cells and RAW 264.7 macrophage‐like cells^###^ were stimulated with the selected preparations for 24 hours and subjected to viability and proliferation assays. The viability measures were determined via formazan formation assay,^13^ Live‐Dead Staining Kit,^14^ and the DNA incorporation of 5‐Bromo‐2′‐Deoxyuridine (BrdU) Cell Proliferation kit.^15^ For caspase‐3 activity, RAW 264.7 macrophage‐like cells were exposed to 30% PRF lysates for 24 hours. Supernatant was removed and the RAW 264.7 cell lysate was exposed to a caspase‐3 substrate following the instructions of the manufacturer.^16^ The RAW 264.7 cell lysate was also assayed for cleaved caspase‐3 (Asp175; 5A1E)^17^ by Western blot.

### 2.5 Statistical analysis

All experiments were repeated at least three times. Statistical analysis was based on Mann‐Whitney *U* test and Kruskal‐Wallis test with Dunn′s multiple comparisons correction. Analyses were performed using a statistical software.^18^ Significance was set at *P* < 0.05.

## 3 RESULTS

### 3.1 PRF maintains viability and increases proliferation of macrophages

**Figure 1:**
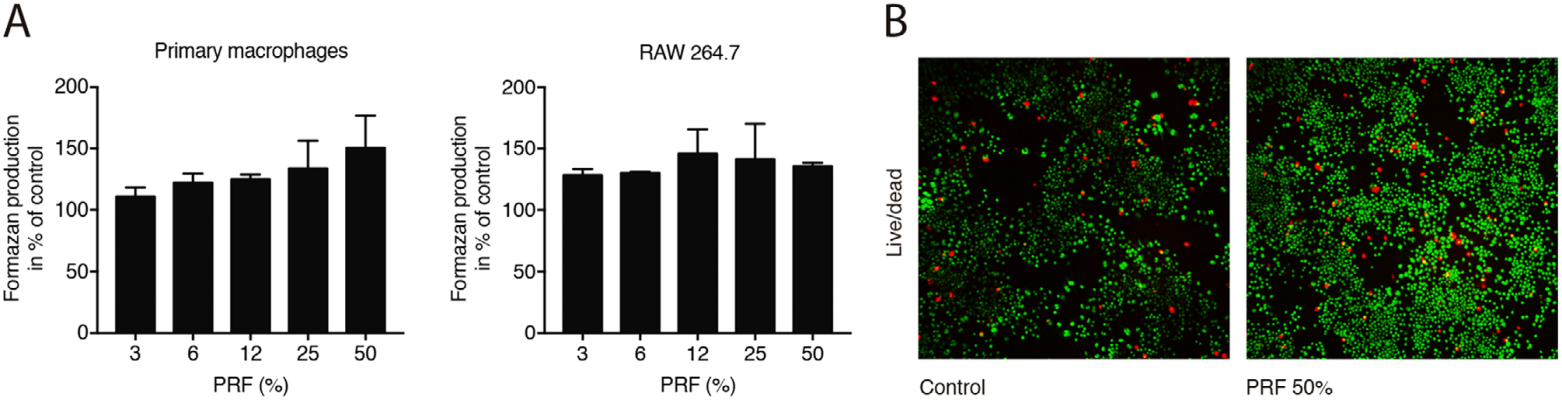
PRF increased metabolic activity of monocyte cells. Primary macrophages and RAW264.7 cells (**A**) were exposed to soluble extracts of PRF membranes at the indicated concentrations for 24 hours. Cell viability is represented by formazan production indicated in percentage of unstimulated controls. (**B**) Live‐Dead staining was performed in RAW264.7 with viable cells appearing in green and dead cells in red. The results from these experiments demonstrated that stimulation with PRF at 50% is highly biocompatible with primary macrophages and RAW264.7. *N* = 4. Data represent the mean ± SD relative to the control

To investigate the impact of soluble extracts of PRF membranes on the cell viability, an MTT assay reflecting the NAD(P)H‐dependent formazan production was carried out. Concentrations below 10% of PRF notably increased formazan production in primary macrophages and RAW264.7 cells (Figure 1A). Further, 30% PRF lysates did not affect the viability of freshly isolated murine bone marrow cells (data not shown). Cell viability was further confirmed by live‐dead staining in RAW264.7 cells (Figure 1B). In addition, PRF further enhanced BrdU incorporation indicating an increased proliferation of RAW264.7 cells (see Supporting Information Table 2 in online *Journal of Periodontology*). Moreover, PRF tended to reduce the expression levels of pro‐apoptotic Bax and caspase‐3 along with the anti‐apoptotic marker gene B cell lymphoma‐2 (BCL2L1) (see Supporting Information Table 3 in online *Journal of Periodontology*). This tendency was further supported by exposing RAW264.7 cells to 30% PRF. PRF lysates suppressed the basal levels of cleaved caspase‐3 (see supplemental Figure 1 in online *Journal of Periodontology*) and caused a weak reduction of caspase‐3 activity (see Supporting Information Figure 1 in online *Journal of Periodontology*). Altogether, these results reveal that soluble extracts of PRF membranes maintain viability and increase proliferation of primary macrophages.

### 3.2 PRF reduces the expression of TRAP and Cathepsin K

**Figure 2:**
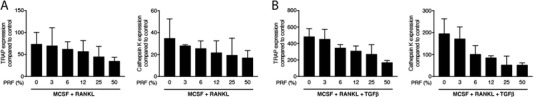
PRF effect is dose‐dependent. Murine bone marrow cells were incubated with various concentrations of soluble extracts of PRF membranes in the presence of RANKL and M‐CSF (**A**) or in the presence of RANKL, M‐CSF and TGF‐β (**B**). Data represent the x‐fold changes in gene expression compared to an MCSF control. *N* = 3. Data are presented as mean ± SD. Statistical analysis was based on Kruskal‐Wallis test with Dunn`s multiple comparisons correction

To determine the most appropriate experimental condition, we evaluated the effect of various concentrations of PRF on gene expression. Murine bone marrow cells were incubated with different concentrations of soluble extracts of PRF membranes in the presence of RANKL and M‐CSF. Dose‐response curves revealed a suppression of the osteoclast marker genes TRAP and Cathepsin K by the addition of PRF (Figure 2A). Next, murine bone marrow cells were incubated with the same concentrations of PRF but in the presence of RANKL, M‐CSF, and TGF‐β. Again, PRF reduced the expression of the osteoclast marker genes TRAP and Cathepsin K in a dose‐dependent manner (Figure 2B). Taken together, these results suggest that 50% of soluble extracts of PRF membranes is a suitable concentration to substantially reduce the aforementioned osteoclast marker genes.

### 3.3 PRF reduces osteoclast differentiation in vitro

**Figure 3:**
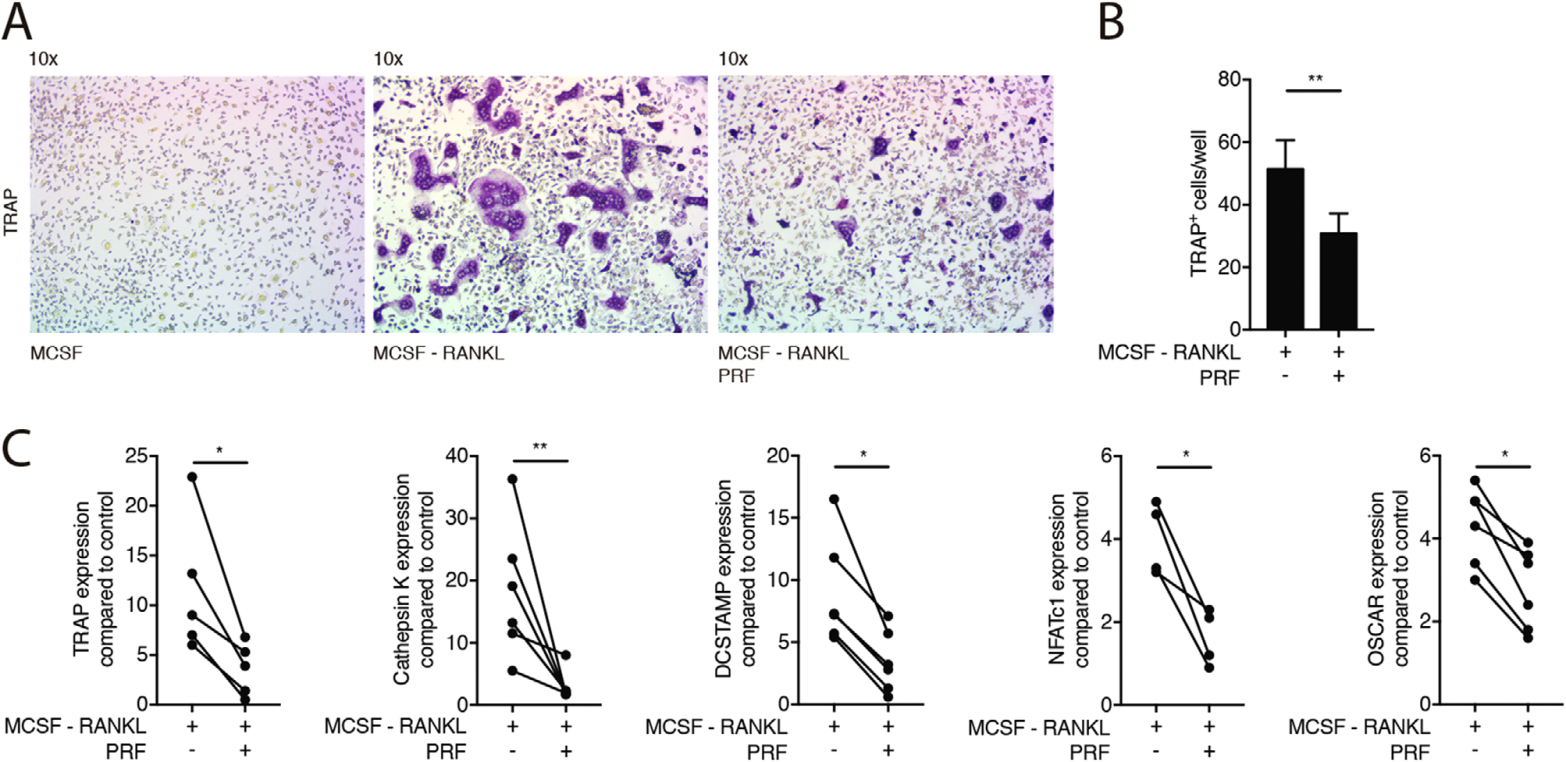
PRF reduces osteoclast differentiation induced by RANKL and M‐CSF. Bone marrow cells were grown in the presence of 50% soluble extracts of PRF membranes to modify osteoclastogenesis induced by RANKL and M‐CSF. (**A**) Representative images of TRAP+ multinucleated osteoclasts in control group (M‐CSF) and in the absence or presence of PRF. (**B**) Mean number ± SD of TRAP+ osteoclasts in absence or presence of PRF. (**C**) Data represent the x‐fold changes in gene expression compared to a M‐CSF control. *N* = 4‐6. Statistical analysis was based on Mann‐Whitney *U* test. Significant changes are indicated by *P < 0.05 and **P < 0.01

To further examine the potential role of PRF in osteoclast differentiation, murine bone marrow cells were grown in the presence of 50% soluble extracts of PRF membranes with RANKL and M‐CSF. We report here that PRF strongly decreased the number of multinucleated cells staining positive for TRAP (Figures 3A and 3B) and also the respective number of nuclei per cells (see Supporting Information Table 4 in online *Journal of Periodontology*). In line with this observation, PRF decreased the expression of TRAP and Cathepsin K, both enzymes required for bone resorption (Figure 3C). In addition, the other osteoclast marker genes DCSTAMP, NFATc1 and OSCAR were also suppressed by soluble extracts of PRF membranes (Figure 3C). Altogether, these observations indicate that PRF can inhibit osteoclastogenesis.

### 3.4 PRF reduces osteoclast differentiation induced by RANKL, M‐CSF, and TGF‐β

**Figure 4:**
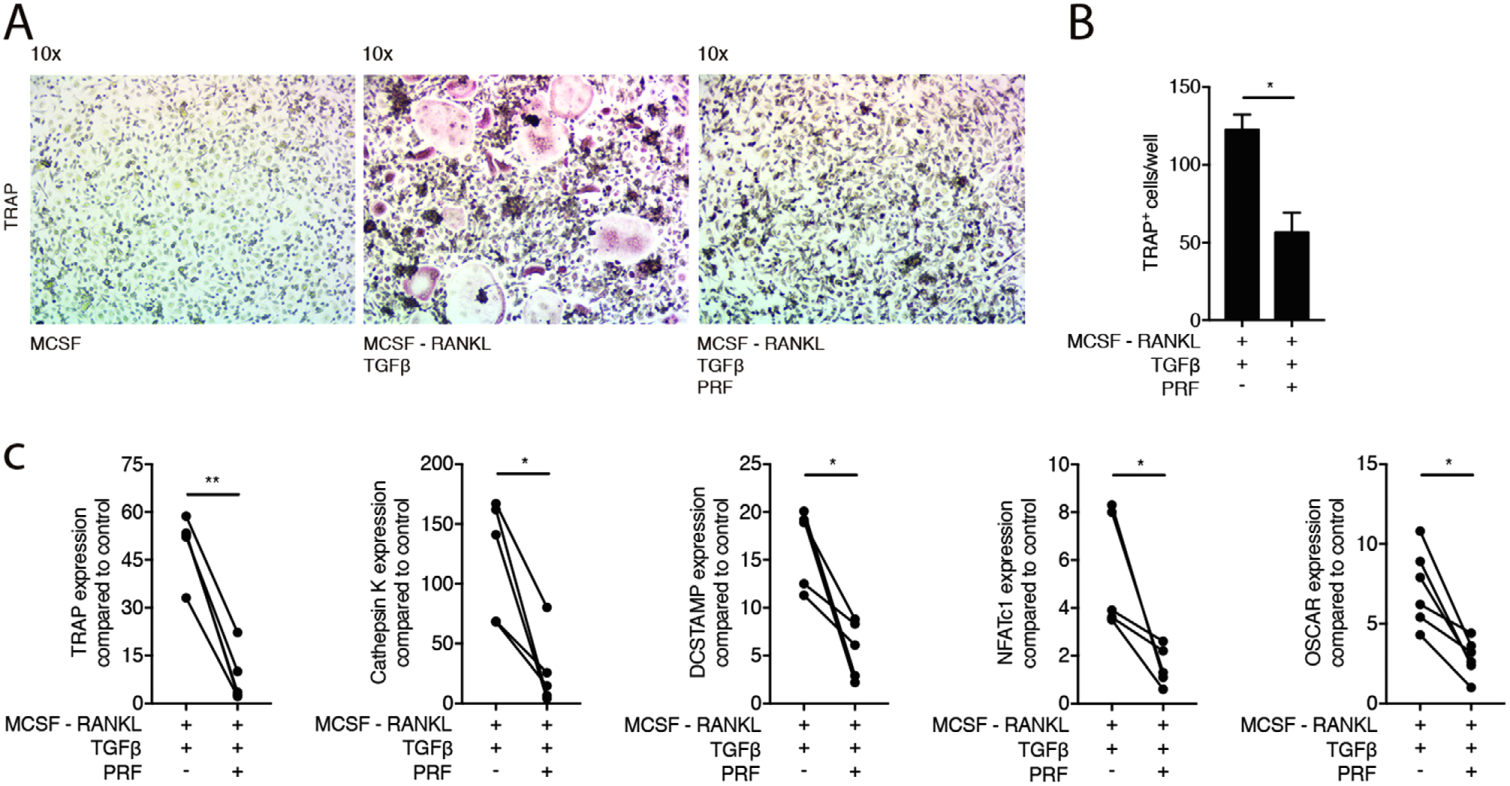
PRF reduces osteoclast differentiation induced by RANKL, M‐CSF and TGF‐β. Bone marrow cells were grown in the presence of 50% soluble extracts of PRF membranes to modify osteoclastogenesis induced by RANKL, M‐CSF and TGF‐β. (**A**) Representative images of TRAP+ multinucleated osteoclasts in the control group (M‐CSF) and in the absence or presence of PRF. (**B**) Mean number ± SD of TRAP+ osteoclasts in absence or presence of PRF. (**C**) Data represent the x‐fold changes in gene expression compared to an M‐CSF control. *N* = 4‐6. Statistical analysis was based on Mann‐Whitney *U* test. Significant changes are indicated by *P < 0.05 and **P < 0.01

To trigger osteoclastogenesis even further, TGF‐β was added to RANKL and M‐CSF as previously described.^20^ As expected, the addition of TGF‐β markedly increased osteoclastogenesis compared to the RANKL and M‐CSF cultures.^20^ Most notably, soluble extracts of PRF membranes at 50% concentration significantly reduced osteoclastogenesis under these conditions as indicated by the reduced number of multinucleated TRAP positive cells (Figures 4A and 4B) and reduced number of nuclei per cells (see Supporting Information Table 4 in online *Journal of Periodontology*). Moreover, PRF lysates decreased the expression of TRAP and Cathepsin K as well as DCSTAMP, NFATc1, and OSCAR (Figure 4C). This suppression of osteoclastogenesis by PRF lysates was further validated by pit formation assay (see Supporting Information Figure 2 in online *Journal of Periodontology*). Taken together, these findings suggest that PRF suppresses osteoclastogenesis independent of the bioassay.

### 3.5 PRF cannot reverse osteoclastogenesis at later stages

**Figure 5:**
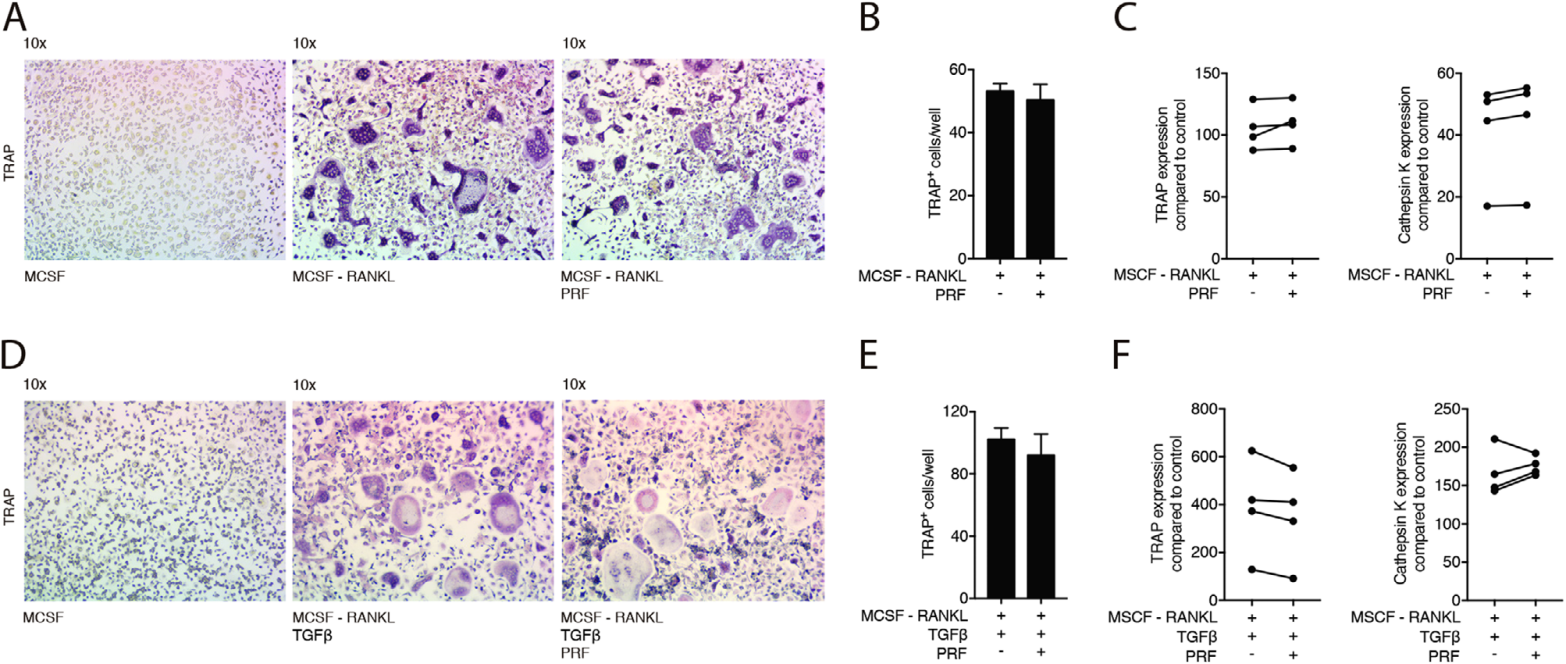
PRF cannot reverse osteoclastogenesis at later stages. Bone marrow cells were grown in the presence of factors M‐CSF, RANKL (**A**) and TGF‐β (**D**). After 72 hours, PRF was added to the cells for another 72 hours. (**A**) and (**D**) Representative images of TRAP+ multinucleated osteoclasts in the control group (M‐CSF) and in the absence or presence of PRF. **(B**) and (**E**) Mean number ± SD of TRAP+ osteoclasts in absence or presence of PRF. (**C**) and (**F**) Data represent the x‐fold changes in gene expression compared to a M‐CSF control. *N* = 4. Statistical analysis was based on Mann‐Whitney *U* test

Considering that PRF reduced the differentiation of osteoclasts from their progenitors, the question then arises whether PRF can reverse this process. To answer this question, murine bone marrow cells were grown in the presence of RANKL and M‐CSF. After 72 hours, PRF was added to the cells for another 72 hours. As shown in Figures 5A and 5D, large multinucleated cells stained for TRAP were observed regardless of whether PRF was added. Furthermore, PRF was not able to reduce the number of osteoclasts (Figures 5D and 5F) neither the numbers of nuclei per cell (data not shown). Moreover, PRF was unable to significantly change the gene expression of the osteoclast marker genes (Figure 5C), also in the presence of TGF‐β (Figure 5F). These findings indicate that PRF is unable to reverse osteoclastogenesis when the process has started.

### 3.6 Growth factors naturally released by PRF decrease osteoclastogenesis

**Figure 6:**
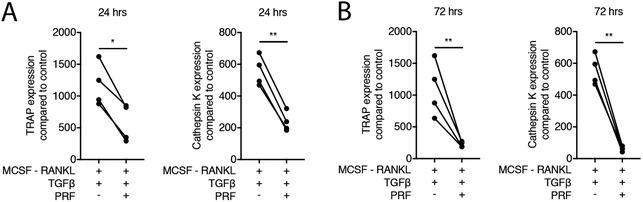
PRF when naturally released decreases osteoclastogenesis. Bone marrow cells were grown in the presence of 50% PRF conditioned medium to change osteoclastogenesis induced by RANKL, M‐CSF and TGF‐β. PRF conditioned medium was harvested after 24 (**A**) and 72 (**B**) hours of incubation at 37°C. Data represent the x‐fold changes in gene expression compared to an M‐CSF control. *N* = 4. Statistical analysis was based on Mann‐Whitney *U* test. Significant changes are indicated by *P < 0.05 and **P < 0.01

Finally, to simulate the natural release of growth factors from PRF membranes, membranes were transferred into culture medium. After 24 and 72 hours the conditioned medium was collected.^18^ Our data show that PRF conditioned medium, independent of the harvesting time, decreased osteoclastogenesis in the presence of RANKL, M‐CSF, and TGF‐β, indicated by TRAP staining (not shown) and the expression of TRAP and Cathepsin K (Figures 6A and 6B). Altogether, these observations suggest that PRF releases an activity that decreases osteoclastogenesis.

## 4 DISCUSSION

This research was inspired by the clinical observation that PRF supports alveolar ridge preservation, suggesting a possible impact of PRF to suppress the formation of bone‐resorbing osteoclasts.^2^, ^3^ Therefore, we induced osteoclastogenesis in vitro using murine bone marrow in the presence of soluble extracts from PRF membranes. The main finding of the present study was that soluble extracts of PRF greatly suppressed the formation of multinucleated cells staining positive for TRAP. In support of these observations, PRF coordinately reduced the respective osteoclast marker genes TRAP and Cathepsin K, as well as the other markers NFATc1, OSCAR, and the gene responsible for cell fusion DCSTAMP. Most notably, PRF inhibited the resorption pit formation. PRF‐conditioned medium also suppressed osteoclastogenesis. Moreover, PRF supported the viability of the macrophages and murine bone marrow cells, and increased the proliferation in the RAW264.7 macrophage cell line; together with a decrease of cleaved caspase‐3. Taken together, we show here that soluble extracts of PRF reduce the formation of osteoclasts originating from bone marrow cells.

If we relate our findings to those of others, our data is consistent with observations that PRF reduced osteoclastogenesis originating from human peripheral blood mononuclear cells.^13^ In contrast to our observations, their decrease was explained by a PRF‐induced apoptosis, similarly to calcium phosphate.^13^ Both studies can therefore not be directly compared as the underlying cause of decreased osteoclastogenesis presumably depends on the model used. Moreover, TRAP is not the exclusive marker for osteoclastogenesis. Thus, we included a panel of target genes that consistently support our main observation of a suppression of osteoclastogenesis by PRF in murine bone marrow cultures. Further and in line with our observations, platelet‐rich plasma inhibits osteoclastogenesis in murine bone marrow cultures.^12^, ^21^ What remains to be determined though are the molecules, contained in PRF, responsible for the inhibition of osteoclastogenesis.

The clinical relevance remains a matter of speculation because it is not clear whether the preservation of the alveolar ridge is a consequence of decreased osteoclastogenesis and bone resorption,^2^, ^3^ a compensatory activation of bone‐forming osteoblasts,^22^ or both. On the other hand, based on the present findings, PRF cannot reverse the process once osteoclasts have developed. Consequently, PRF cannot turn mature osteoclasts back into an undifferentiated macrophage‐like phenotype. In this sense, it remains unknown whether these findings can be translated into a clinical scenario such as alveolar ridge preservation. The inhibition of osteoclasts differentiation by PRF not necessarily implies a decrease in bone resorption. In addition, the limited suppression on osteoclasts by PRF at later stages might not represent the in vivo situation. Nevertheless, these observations provide new insights into a plausible mechanism of PRF that may support a clinical benefit of PRF.

Another interesting aspect that requires further attention is the possible benefits of using PRF in conjunction with other biomaterials including dental implants. A recent systematic review by our group revealed that PRF increases the implant stability during the early phases of osseointegration. Because dental implants decrease the expression of osteoclast markers in cortical osteotomy defects after one month of healing,^23^ the use of PRF may further decrease osteoclastogenesis leading to a higher implant stability during the first months.^4^ Moreover, during GBR procedures a variety of other biomaterials are routinely used such as collagen membranes. Collagen membranes can cause immigration of macrophages,^24^ which might not necessarily become osteoclasts.^25^ Antagonizing this process by the addition of PRF could potentially inhibit osteoclastogenesis under inflammatory conditions thereby enhancing biomaterial integration.

The study has limitations. First, as long as the molecular and cellular mechanisms causing resorption of the alveolar bone, particularly the bundle bone, have not been elucidated, and our knowledge depends on clinical observations and descriptive histology,^26^ a targeted therapy to prevent the alveolar ridge from resorption cannot be developed. We cannot rule out that, apart from reducing osteoclastogenesis, PRF might, for example, preserve the viability of osteocytes and thereby hinder the release of osteoclast‐activating signals.^27^ Second, we have used a xenogenic approach using murine bone marrow and human PRF. It is, however, challenging to generate sufficient PRF from mice as the blood volume is low and using bone marrow cells from humans reaches the borders of ethical acceptance. Additionally, the donor heterogeneity might lead to a high variation reducing the power of the analysis. Finally, we have not considered the impact of centrifugation force and time leading to different PRF‐based matrices on osteoclastogenesis.^28^ We decided to use the same protocol and hardware to minimize the number of variables and achieve reproducibility in accordance to previous recommendations.^29^ The decrease in osteoclastogenesis might be associated with the M1‐to‐M2 shift of macrophages exposed to PRF lysates.^30^ Future research should focus on the molecular mechanism by which PRF inhibits osteoclastogenesis not only in vitro, but also in vivo and whether different PRF protocols modulate osteoclastogenesis differently. The most suitable preparation of PRF to inhibit osteoclastogenesis and obtain the best clinical outcomes in ridge preservation remains unclear.^3^, ^4^ Moreover, because different PRF protocols produce different amounts of growth factors and consequently biological responses,^28^ further research should be undertaken to determine the best setting for each protocol to obtain the most predictable clinical results.

## 5 CONCLUSION

Taken together, these in vitro findings suggest that (i) PRF suppresses osteoclastogenesis and (ii) PRF cannot reverse osteoclastogenesis once osteoclasts have developed. From a clinical point of view our results imply that favorable effects of PRF membranes in alveolar ridge preservation may be attributed, at least in part, by the inhibition of osteoclastogenesis.

## AUTHOR CONTRIBUTIONS

Reinhard Gruber and Franz Josef Strauss contributed to conception and design; contributed to acquisition, analysis, and interpretation; drafted manuscript; critically revised manuscript; gave final approval; agreed to be accountable for all aspects of work. Zahra Kargarpour and Jila Nasirzade contributed to conception and design, contributed to acquisition, analysis, and interpretation; critically revised manuscript; gave final approval; agreed to be accountable for all aspects of work. Francesca Di Summa, Heinz‐Dieter Müller, and Sadegh Hasannia contributed to acquisition, analysis, and interpretation; critically revised manuscript; gave final approval; agreed to be accountable for all aspects of work.

## Supporting information


**Table 1**: Primer sequences.

**Table 2**: PRF increased proliferation of monocyte cells.

**Table 3**: PRF‐related expression of apoptosis markers in bone marrow macrophages.

**Table 4**: PRF reduces the number of nuclei per TRAP positive osteoclast.

## Footnotes

1 BD Vacutainer Ref 367896; BD, Plymouth, UK.

2 Z 306 Hermle Universal Centrifuge, Wehingen, Germany.

3 Sonopuls 2000.2, Bandelin electronic, Berlin, Germany.

4 Sigma Aldrich, St. Louis, MO.

5 Bio&SELL GmbH, Nuremberg, Germany.

6 ProSpec, Ness‐Ziona, Israel.

7 Sigma‐Aldrich, Germany.

8 ExtractMe, Blirt S.A., Gdańsk, Poland.

9 SensiFAST, Bioline Reagents Ltd., London, UK.

10 CFX Connect, BioRad, Hercules, CA.

11 CFX Maestro™, BioRad, Hercules, CA.

12 BioRad, Hercules, CA.

13 Sigma, St. Louis, MO.

14 Lausen, Switzerland.

15 Roche Life Science, Penzberg, Germany.

16 Novus Biologicals, Centennial, CO.

17 Cell Signaling Technology Europe, B.V., Frankfurt am Main, Germany.

18 GraphPad Software, La Jolla, CA.
