# Supplementary material for: Platelet‐rich fibrin suppresses in vitro osteoclastogenesis
Source: J Periodontol. 2019 Sep 17;91(3):413–21. doi: 10.1002/JPER.19-0109 (PMC7155126; doi:10.1002/JPER.19-0109)
Supplement: Supplementary file 1 — Table 1: Primer sequences. [file JPER-91-413-s001.docx]

*Table 1: Primer sequences*

Gene Sequence forward Sequence reverse

m GAPDH aac ttt ggc att gtg gaa gg gga tgc agg gat gat gtt ct

m cathepsin-K tgt ata acg cca cgg caa a ggt tca cat tat cac ggt cac a

m DC-Stamp aag ctc ctt gag aaa cga tca cag gac tgg aaa cca gaa atg

m TRAP aag cgc aaa cgg tag taa gg cgt ctc tgc aca gat tgc at

m OSCAR agt cca agg agc cag aac ct agt cca agg agc cag aac ct

m NFATc-1 ccg ttg ctt cca gaa aat aac a tgt ggg atg tga act cgg aa

m BCL2L1 tga cca cct aga gcc ttg ga gag ggg tgt acc tcc act ca

m Bax agt gtc tcc ggc gaa ttg cca cgt cag caa tca tcc t

m Caspase3 gag gct gac ttc ctg tat gctt aac cac gac ccg tcc ttt
