# Supplementary material for: Platelet‐rich fibrin suppresses in vitro osteoclastogenesis
Source: J Periodontol. 2019 Sep 17;91(3):413–21. doi: 10.1002/JPER.19-0109 (PMC7155126; doi:10.1002/JPER.19-0109)
Supplement: Supplementary file 2 — Table 2: PRF increased proliferation of monocyte cells. [file JPER-91-413-s002.docx]

*Table 2: PRF increased proliferation of monocyte cells*

RAW264.7 cells were exposed to PRF membranes at the indicated concentrations for 72 hours. Cell proliferation was determined by DNA incorporation of BrdU, presented as percentage of unstimulated controls.

| Nº Experiment | PDGF-BB | PRF 50% | PRF 25% | PRF 12% |
| --- | --- | --- | --- | --- |
| Experiment 1 | 114.4 | 119.6 | 115.4 | 104.2 |
| Experiment 2 | 104.6 | 121.1 | 112.3 | 113.3 |
