# Supplementary material for: Platelet‐rich fibrin suppresses in vitro osteoclastogenesis
Source: J Periodontol. 2019 Sep 17;91(3):413–21. doi: 10.1002/JPER.19-0109 (PMC7155126; doi:10.1002/JPER.19-0109)
Supplement: Supplementary file 3 — Table 3: PRF‐related expression of apoptosis markers in bone marrow macrophages. [file JPER-91-413-s003.docx]

*Table 3: PRF-related expression of apoptosis markers in bone marrow macrophages*

Primary macrophages were exposed to RANKL and M-CSF, with and without 50% PRF lysates for 6 days. Expression of apoptosis marker genes is expressed as x-fold change of RANKL and M-CSF controls.

| N° Experiment | Bax | BCL2L1 | Caspase-3 |
| --- | --- | --- | --- |
| Experiment 1 | 0.9 | 0.9 | 1.3 |
| Experiment 2 | 0.9 | 0.8 | 1.4 |
| Experiment 3 | 0.7 | 0.7 | 0.8 |
| Experiment 4 | 1.0 | 1.0 | 0.6 |
