# Supplementary material for: Platelet‐rich fibrin suppresses in vitro osteoclastogenesis
Source: J Periodontol. 2019 Sep 17;91(3):413–21. doi: 10.1002/JPER.19-0109 (PMC7155126; doi:10.1002/JPER.19-0109)
Supplement: Supplementary file 4 — Table 4: PRF reduces the number of nuclei per TRAP positive osteoclast. [file JPER-91-413-s004.docx]

*Table 4: PRF reduces the number of nuclei per TRAP positive osteoclast*

Murine bone marrow cells were grown in the presence of 50% PRF lysates with RANKL, M-CSF and with or without TGF-β. The nuclei of each TRAP positive cell from three random fields were manually counted Data represent mean ± SD.

| Nº Experiment | M-CSF + RANKL | M-CSF + RANKL + PRF | M-CSF + RANKL + TGF-β | M-CSF + RANKL +TGF-β + PRF |
| --- | --- | --- | --- | --- |
| Experiment 1 | 3.0 $\pm$ 1.7 | 1.3 $\pm$0.7 | 13.8 $\pm$3.2 | 1.1 $\pm$ 0.3 |
| Experiment 2 | 3.9 $\pm$ 1.1 | 1.4 $\pm$0.8 | 10.2 $\pm$ 4.0 | 1.3 $\pm$0.6 |
